# The Therapeutic Potential of Carnosine/Anserine Supplementation against Cognitive Decline: A Systematic Review with Meta-Analysis

**DOI:** 10.3390/biomedicines9030253

**Published:** 2021-03-04

**Authors:** Giuseppe Caruso, Justyna Godos, Sabrina Castellano, Agnieszka Micek, Paolo Murabito, Fabio Galvano, Raffaele Ferri, Giuseppe Grosso, Filippo Caraci

**Affiliations:** 1 Department of Drug and Health Sciences, University of Catania, 95125 Catania, Italy; forgiuseppecaruso@gmail.com (G.C.); f.caraci@unict.it (F.C.); 2 Department of Biomedical and Biotechnological Sciences, University of Catania, 95123 Catania, Italy; justyna.godos@gmail.com (J.G.); fgalvano@unict.it (F.G.); 3 Department of Educational Sciences, University of Catania, 95124 Catania, Italy; sabrina.castellano@unict.it; 4 Department of Nursing Management and Epidemiology Nursing, Faculty of Health Sciences, Jagiellonian University Medical College, 31-501 Krakow, Poland; agnieszka.micek@uj.edu.pl; 5 Department of General Surgery and Medical-Surgical Specialties, Section of Anesthesia and Intensive Care, University of Catania, 95123 Catania, Italy; paolo.murabito@unict.it; 6 Oasi Research Institute—IRCCS, 94018 Troina, Italy; rferri@oasi.en.it

**Keywords:** carnosine, cognitive function, depressive symptoms, age-related cognitive decline, Alzheimer’s disease, neuroinflammation, oxidative stress

## Abstract

Carnosine is a natural occurring endogenous dipeptide that was proposed as an anti-aging agent more than 20 years ago. Carnosine can be found at low millimolar concentrations at brain level and different preclinical studies have demonstrated its antioxidant, anti-inflammatory, and anti-aggregation activity with neuroprotective effects in animal models of Alzheimer’s disease (AD). A selective deficit of carnosine has also been linked to cognitive decline in AD. Different clinical studies have been conducted to evaluate the impact of carnosine supplementation against cognitive decline in elderly and AD subjects. We conducted a systematic review with meta-analysis, in accordance with the PRISMA guidelines coupled to the PICOS approach, to investigate the therapeutic potential of carnosine against cognitive decline and depressive symptoms in elderly subjects. We found five studies matching the selection criteria. Carnosine/anserine was administered for 12 weeks at a dose of 1 g/day and improved global cognitive function, whereas no effects were detected on depressive symptoms. These data suggest a preliminary evidence of clinical efficacy of carnosine against cognitive decline both in elderly subjects and mild cognitive impairment (MCI) patients, although larger and long-term clinical studies are needed in MCI patients (with or without depression) to confirm the therapeutic potential of carnosine.

## 1. Introduction

Carnosine is a natural occurring endogenous dipeptide discovered by Gulewitsch and Amiradžibi during the analysis of a meat extract more than 100 years ago [1]. The synthesis of this molecule starting from its constituting amino acids, β-alanine and L-histidine, through an enzyme-catalyzed reaction requiring Mg^++^ and adenosine triphosphate (ATP) was first described in 1950s [2,3]. Carnosine has been found in the tissues and organs of vertebrates [4] as well as in the tissues of some invertebrate species [5,6]. With regard to mammalian, this widely distributed molecule is present in different organs, such as spleen and kidney [7], and can be found at low millimolar (mM) concentrations at brain level [8], while it reaches high mM concentrations (up to 20 mM) in cardiac and skeletal muscles [9]. In a study by Fonteh et al., a selective deficit of carnosine has been related to cognitive decline in probable Alzheimer’s disease (pAD) subjects [10]. In this study, where the free amino acid and dipeptide changes in the body fluids from pAD subjects were analyzed, carnosine levels were significantly lower in pAD (328.4 ± 91.31 nmol/dl) than in plasma of healthy subjects (654.23 ± 100.61nmol/dl); this deficit of carnosine correlated with reduced global cognitive function measured by Mini-Mental State Examination (MMSE) and Alzheimer’s Disease Assessment Scale cognitive subscale (ADAS-cog).

The decrease of carnosine levels in AD is also favored by the age-related increase in the serum-circulating (CNDP1 or CN1) activity in specific brain regions [11]. In fact the concentrations of carnosine in human tissues and biological fluids are regulated by the activity of two dipeptidases: CNDP1 [12] and the cytosolic (CNDP2 or CN2) carnosinase [13], both of them members of the M20 metalloprotease family [14]. As it has been shown for the first time by Perry and colleagues, patients with carnosinase deficiency, a condition also known as carnosinemia, present an excess of carnosine in the urine (carnosinuria) and develop a progressive neurologic disorder characterized by severe mental defects and intellectual disability [15,16,17].

Different mechanisms have been identified that can explain the hypothesized protective activity of carnosine against cognitive decline [18]. In fact, it can act as neurotransmitter [19], immune system enhancer [20], nitric oxide metabolism modulator [21,22], heavy metal chelator [23,24], cell energy metabolism enhancer [25,26], anti-glycation, and anti-aging agent [27,28]. Carnosine has also been shown to modulate glutamatergic system by upregulating the glutamate transporter 1 and reducing glutamate concentrations in the central nervous system (CNS) [29].

It is becoming increasingly evident that neuroinflammation [30,31,32] and oxidative stress [33,34], along with the abnormal accumulation of proteins at brain level [35,36], significantly contribute to the cognitive decline associated to different pathologies of CNS. According to this scenario, the well-known antioxidant, anti-inflammatory, and anti-aggregation activities of carnosine have been recently reconsidered [37], to better understand the therapeutic potential of this peptide in the treatment of cognitive disorders. In a preclinical study by Herculano et al., the treatment with carnosine (5 mg/day for six weeks) was able to rescue cognitive deficits and revert oxidative stress and microglial activation induced by an high fat diet in the hippocampus of a transgenic mouse model of AD [38]. The rescue of cognitive deficit by carnosine was also demonstrated in streptozotocin-induced diabetic rats [39], as well as in subcortical ischemic vascular dementia [40] and transgenic 3 × Tg-AD mice, showing both amyloid beta (Aβ)- and tau-dependent pathology [41]. Preclinical studies in mice have demonstrated that this dipeptide is essentially non-toxic [42]; additionally, it is well tolerated in humans [43,44] without known drug interactions and dangerous side effects.

Moving from mice to humans, different clinical trials have been conducted to explore the therapeutic effects of carnosine in cognitive disorders. In a randomized double-blind placebo controlled 12-week dose escalation study, involving 25 Gulf War illness subjects, carnosine (1500 mg/day) gave beneficial cognitive effects [45]. In a study carried out by Masuoka et al., the potential protective effects of anserine/carnosine (3:1) supplementation against cognitive decline in APOE4 (+) mild cognitive impairment (MCI) subjects were shown, possibly by preventing a transition from MCI to AD [46]. Improvements on cognitive functioning have also been observed in two different studies, using a pill-based nutraceutical (NT-020) containing carnosine in older adults [47] or a formulation (formula F) including carnosine administered along with donepezil to moderate probable AD subjects [48].

Carnosine was proposed as an anti-aging agent more than 20 years ago [28]. During the following years, several human studies have been carried out to test its possible positive effects in the elderly. It has been shown that dietary supplementation of carnosine (250–350 mg/daily) in combination with its methylated analogue anserine (β-alanyl-1-N-methyl-L-histidine) (650–750 mg/daily) for about 13 weeks is able to improve cognitive function [49,50] and physical activity [50], to preserve verbal episodic memory and brain perfusion [49,51], and to modulate network connectivity changes associated with cognitive function [49] in elderly people.

Despite numerous preclinical and clinical studies that have been carried out, the effects of carnosine supplementation in preventing and/or counteracting cognitive decline in humans have not yet been completely understood. Recent reviews provide a comprehensive overview of the role of carnosine in neurological, neurodegenerative, and psychiatric disorders [52], although no specific meta-analyses have been conducted to analyze the clinical efficacy of carnosine in different double-blind randomized placebo-controlled trials.

With the present study, we aimed to address this specific gap in the knowledge of the therapeutic potential of carnosine against cognitive decline by conducting a systematic review with meta-analysis, in accordance with the PRISMA guidelines coupled to the PICOS approach, to investigate the effects of this peptide with a multimodal pharmacodynamic profile on cognitive function and depressive symptoms in elderly subjects.

## 2. Methods

The design, analysis, and reporting of this study followed Preferred Reporting Items for Systematic Reviews and Meta-Analysis (PRISMA) (Supplementary Table S1) [53]. Moreover, eligibility criteria for the search and meta-analyses were specified using the PICOS approach: Determination of the population (P), intervention (I), comparison (C), outcomes (O), study design (S) (Table 1).

### 2.1. Study Selection

A systematic search on PubMed (http://www.ncbi.nlm.nih.gov/pubmed/ (accessed on 4 March 2021)), EMBASE (http://www.embase.com/ (accessed on 4 March 2021)), and Web of Science (www.webofknowledge.com (accessed on 4 March 2021)) databases of studies published up to April 2020 was performed using the following search strategy: (carnosine OR l-carnosine OR n-acetyl-carnosine OR n-acetyl-l-carnosine OR histidine OR beta-alanyl-l-histidine OR b-alanyl-l-histidine OR L-Histidine OR l-alpha-alanyl-l-histidine OR beta-alanine OR β-alanine OR beta alanyl 3 methylhistidine OR 3-aminopropionic-acid OR anserine) AND (cognitive OR cognition OR mental OR mood OR memory OR learning OR attention OR depression OR depressive OR schizophrenia OR Alzheimer OR Alzheimer’s OR autism OR sleep) AND (randomized clinical trial OR controlled clinical trial OR randomized OR placebo OR clinical trial OR trial OR randomly OR intervention OR enrolled). The search was restricted to the studies conducted in humans. Studies were eligible if they met the following inclusion criteria: (i) were intervention studies with control group; (ii) were conducted on adults; (iii) evaluated the effect of carnosine supplementation on cognitive function and/or depression; (iv) evaluated long term effects of carnosine rather than acute effects. Studies which evaluated the effect of carnosine on cognitive performance were excluded. Finally, the studies that provided sufficient statistical data were further considered for the meta-analysis. Reference lists of eligible studies were scanned for any additional study not previously identified. If more than one study reported results on the same individuals, only the study including the larger sample size, the longest follow-up, or the most comprehensive statistical data was included in the meta-analysis. The systematic search and study selection was performed by two independent authors (J.G. and G.C.).

### 2.2. Data Extraction

Data were extracted using a standardized extraction form. The following information was collected: (i) First author name and publication year; (ii) study design and country; (iii) sample size and intervention duration; (iv) sex and mean age of participants; (v) type of intervention and its main characteristics; (vi) outcome scores, including means and standard deviations or standard errors or 95% confidence intervals (Cis) for each score at baseline and after follow-up for each group (intervention and control) or *p*-value for significance of this change (from paired t-test or Wilcoxon test).

### 2.3. Study Quality and Risk of Bias Assessment

The quality of each eligible study was assessed according to the NIH Quality Assessment of Controlled Intervention Studies (Supplementary Table S2). This tool allows the rater to assign a three-level quality score (“good”, “fair”, or “poor”), based on the consideration of 14 items. The tool for controlled intervention studies evaluates the following: adequate randomization, treatment allocation and blinding, similarity of groups at the baseline, dropout rates, adherence to the treatment, avoidance of other interventions, outcome measures assessment, sample size and power calculation, pre-specified outcomes, and intention-to-treat analysis. Risk of bias across included studies was assessed using Cochrane risk-of-bias tool for randomized trials (RoB-2) [54].

### 2.4. Statistical Analysis

All studies identified during systematic search had the independent-group pre-test–post-test design (i.e., the outcome was measured before and after intervention, and different groups received the experimental intervention or served as control group) [55]. We used standardized mean difference (SMD) due to necessity of harmonizing different scores measuring the same outcome with different tools. We used the so-called raw score metric to focus on group differences (i.e., the effect size for SMD in each intervention group was defined as the pre-test–post-test change divided by the pre-test standard deviation, due to likeness being more consistent across studies as not being influenced by the experimental manipulations) [56]. Transforming effect sizes into raw score metric required an estimate of the population correlation between pre- and post-test, which was [55]. Fixed-effects models were used to perform all meta-analyses irrespectively of heterogeneity due to small number of trials. In order to compare tools with differences in the direction of the scale, the mean values from one set of studies was multiplied by −1. Finally, in order to test whether variation between studies in effect size was associated with differences in methodology of the studies or in characteristics of participants, meta-regression analyses were performed taking into account age, sex, sample size, length of trial, and baseline scores value. A two-sided *p*-value 0.05 was set as the level of significance for comparisons of SMD. Data were analyzed using R software version 3.6.1 (Development Core Team, Vienna, Austria).

## 3. Results

### 3.1. Study Identification and Selection Process

**Figure 1:**
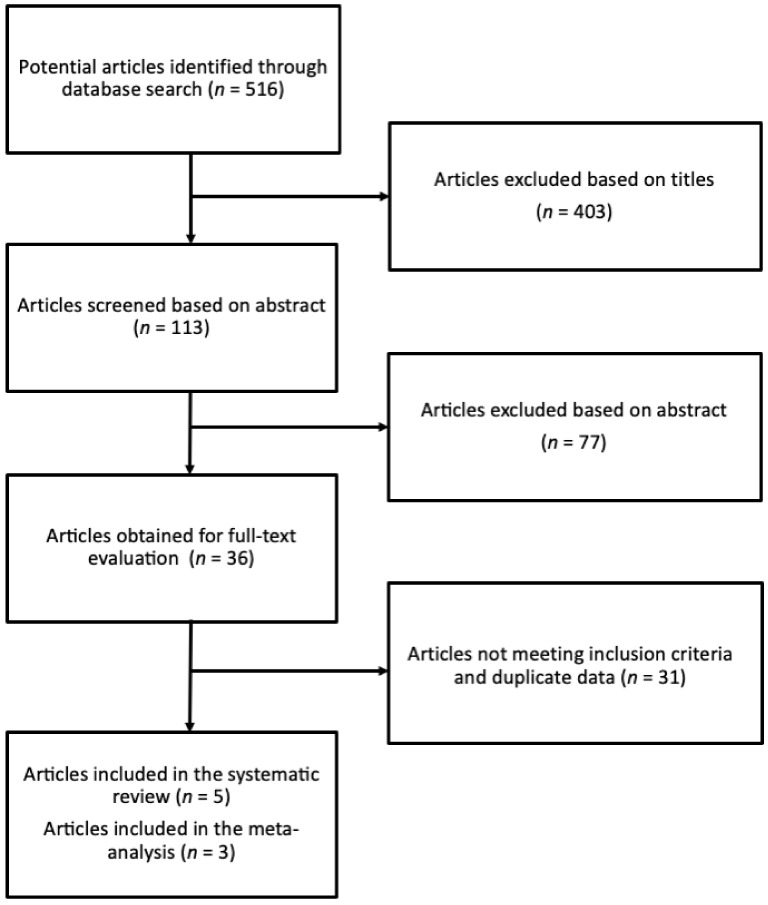
Study selection process.

The systematic search yielded 516 studies, out of which 403 and 77 were excluded based on the title and abstract evaluation, respectively. Thirty-six articles were assessed based on the full-text version, and 31 studies did not meet the pre-specified inclusion criteria. In particular, the studies were excluded as they (i) reported on the acute effects of carnosine supplementation, (ii) reported results on children, (iii) reported on other outcomes, such as quality of life of cognitive performance, (iv) did not explore the outcomes of interest, and (v) were conducted on partially same patients (thus, only the latest report was included). Finally, five studies [46,48,50,57,58] were included in the systematic review, out of which three provided sufficient statistical data and were included in the quantitative analysis (Figure 1).

The main characteristics of the studies included in the systematic review and meta-analysis are presented in Table 2.

One study was conducted in USA [48], three in Asia [46,57,58], and one in Europe [50]. The outcome measures explored in the studies included Mini Mental State Examination (MMSE) [46,48,50,57], Alzheimer’s Disease Assessment Scale (ADAS) [46,57], Clinical Dementia Rating (CDR) [46,50], Geriatric Depression Scale (GDS) [46,50], Beck Depression Inventory (BDI) [57], Wechsler Memory Scale Logical Memory (WMS-LM) [46,49], Profile of Mood Scale (POMS) [58], Mental health Component Summary (MCS) [57], and Short Test of Mental Status (STMS) [50]. Sample size and trial length ranged from 48 to 72 individuals and six to 13 weeks, respectively.

### 3.2. Study Quality Assessment

Based on the NIH Quality Assessment of Controlled Intervention Studies, four of the studies reached a “good” quality score, while one scored as “fair” quality. The main limitation was that the studies did not use intention-to-treat analysis and in several cases the sample size was not sufficiently large to be able to detect a difference in the main outcome between groups with at least 80% power.

### 3.3. Risk of Bias

**Figure 2:**
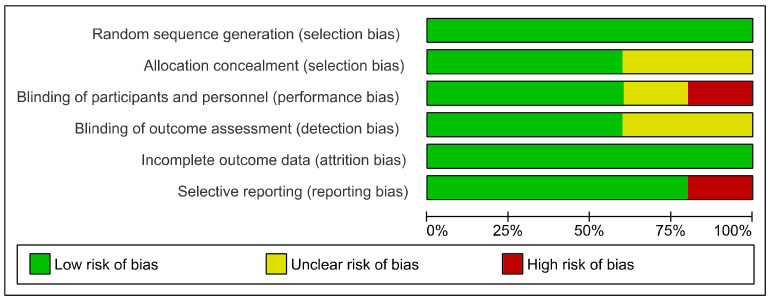
Summary of the risk of bias assessment according to the Cochrane risk-of-bias tool for randomized trials (RoB-2).

According to the Cochrane RoB-2, most of the studies had low risk of selection and attrition bias (Figure 2, Supplementary Figure S1).

However, one study demonstrated high risk of performance bias due to the lack of blinding [58], and one study high reporting bias as some of the data were not reported in the manuscript [48].

### 3.4. Carnosine Supplementation and Cognitive and Memory Function

Four individual studies explored whether carnosine supplementation did affect cognitive function [46,48,50,57]. The study of Cornelli [48] involved 52 patients (mean age about 75 years old) affected with moderate probable Alzheimer’s disease already being treated with donepezil (5 mg/day for at least two months); the authors reported that the MMSE remained stable in the group treated with standard therapy and placebo, while significant improvements were found in the intervention group with donepezil plus a formula containing 100 g of carnosine (among other antioxidants). In the study of Szcześniak et al. [50], 56 healthy subjects (age 65+ years old) were administered chicken meat extract containing 40% of anserine and carnosine components or a placebo for a 13 weeks of supplementation; the mean values of the Short Test of Mental Status (STMS) scores showed a significant increase in the intervention group, specifically in the sub scores of construction/copying, abstraction, and recall. The study of Katakura et al. [57] involved 60 healthy elderly volunteers administered 1 g carnosine/anserine or a placebo for three months; preservation of verbal memory, assessed by the WMS-LM, was observed in the intervention group (especially among older participants), while no significant changes were observed in other cognitive function measures. A significant correlation was also found between the preservation of verbal memory and suppression of C-C Motif Chemokine Ligand 24 (CCL24; an inflammatory chemokine) expression in the group that was in their 70s. The last published trial from Masouka et al. [46] on 54 subjects with MCI, randomized to an active group receiving a dose of 1 g per day of carnosine/anserine or a placebo for 12 weeks, showed improvement in the global Clinical Dementia Rating in the active group, as compared for placebo, but no significant results in the other psychometric tests, including the MMSE and the ADAS. The authors did not detect improvements in verbal episodic memory, but, interestingly, when they separated APOE4 positive (APOE4 (+)) or negative (APOE4 (−)) subjects, a clinically-relevant change was observed in the APOE4 (+) subjects both in MMSE and in gloCDR scores.

**Figure 3:**
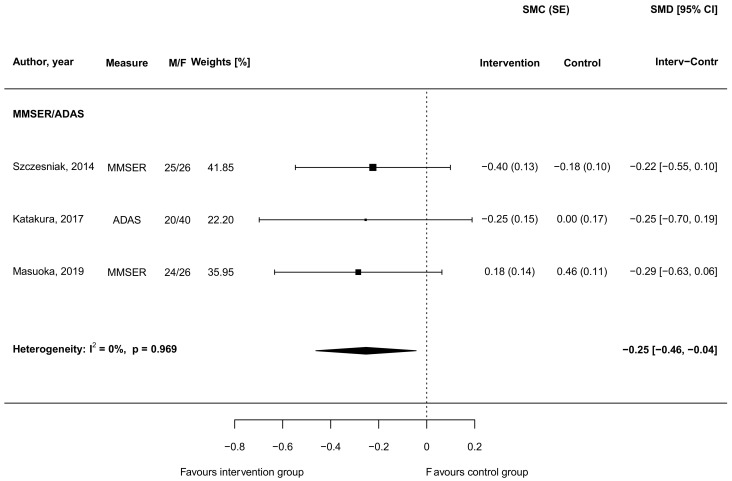
Standardized mean differences in cognitive outputs between intervention groups supplemented with carnosine and control groups in randomized controlled trials. Abbreviations: ADAS (Alzheimer’s Disease Assessment Scale), CI (confidence interval), F (female), M (male), MMSER (Mini Mental State Examination), SE (standard error), SMC (standardized mean change), SMD (standardized mean difference).

Out of the four studies, three provided sufficient data to be eligible for quantitative comparison of cognitive outcomes. The meta-analysis included two studies testing cognitive function through the MMSE [46,50] and one through the ADAS tool [57]. Although the results from individual studies did not show significant differences between intervention and control groups, results from the meta-analysis (presented in Figure 3) revealed that supplementation with carnosine led to a SMD of -0.25 (95% CI = −0.46, −0.04) in favor of the intervention compared to the control group, indicating an improvement in cognitive function.

No evidence of heterogeneity between studies (I2 = 0%, *p* = 0.969) nor asymmetry on funnel plots (Supplementary Figure S2) were found.

Meta-regression analyses were conducted to test whether study-related characteristics may have affected the results; however, none of the other variables investigated as moderators substantially influenced the overall effect size (Supplementary Table S3).

**Figure 4:**
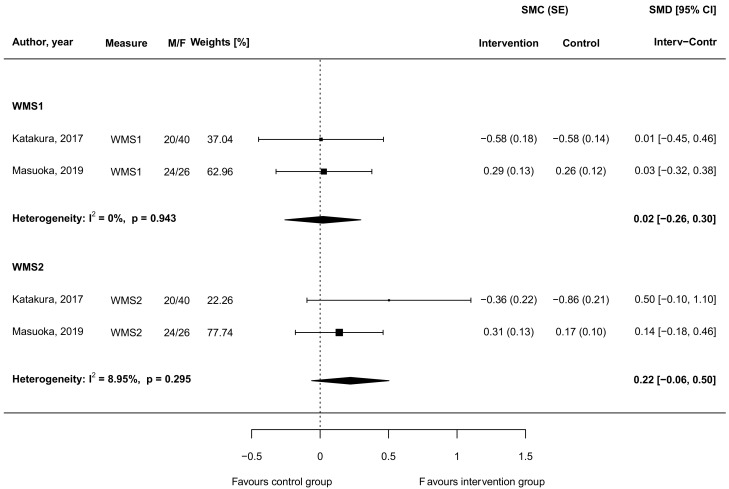
Standardized mean differences in verbal memory outputs between intervention groups supplemented with carnosine and control groups in randomized controlled trials. Abbreviations: CI (confidence interval), F (female), M (male), SE (standard error), SMC (standardized mean change), SMD (standardized mean difference), WMS (Wechsler Memory Scale).

Results from the two studies reporting comparable quantitative data on verbal memory, assessed through the Wechsler memory scale–revised logical memory immediate recall (WMS-LM1) and delayed recall (WMS-LM2) tests, are shown in Figure 4.

While nearly no differences could be observed for the WMS-LM1, an improvement, yet not significant, in the WMS-LM2 was found in the intervention compared to the control group (SMD = 0.22, 95 CI: −0.06, 0.50), with no evidence of heterogeneity between studies (I2 = 8.95%, *p* = 0.295; Figure 4).

All studies but one lasted less than 12 weeks, thus no meta-regression was conducted on length of trial. None of the other variables investigated as moderators substantially influenced the overall effect size (Supplementary Table S3).

### 3.5. Carnosine Supplementation and Depressive Symptoms

**Figure 5:**
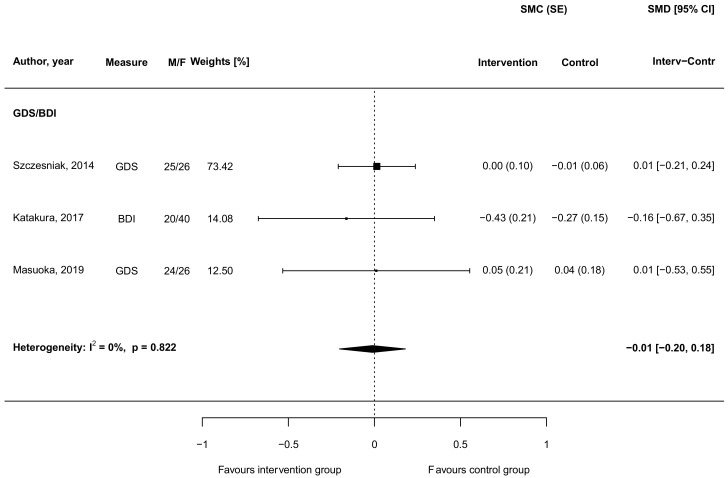
Standardized mean differences in depressive symptoms outputs between intervention groups supplemented with carnosine and control groups in randomized controlled trials. Abbreviations: BDI (Beck Depression Inventory), CI (confidence interval), F (female), GDS (Geriatric Depression Scale), M (male), SE (standard error), SMC (standardized mean change), SMD (standardized mean difference).

The effect of carnosine supplementation toward depressive symptoms was investigated in three studies [46,50,57]. One of these studies reported no improvement of depressive symptoms among all subjects measured with the GDS, also authors did not observe any altering of the distribution of ratings of depressive symptoms in the carnosine supplemented individuals [50]. Additionally, Masuoka et al. reported null results when considering depressive symptoms as an outcome [46]. However, in another study, a weak trend towards improvement in the BDI test for assessing the level of depression following three-month supplementation was observed [57]. When pooling together results from all investigations, no significant results were found (SMD = −0.01, 95% CI: −0.20, 0.18; Figure 5).

No findings are to be reported concerning meta-regression analysis on the role of potential moderators on results (Supplementary Table S3).

### 3.6. Carnosine Supplementation and Mood

Solely one eligible study explored the effect of carnosine supplementation on mood (measured using POMS questionnaire). The study enrolled 72 healthy full-time office workers and randomized them into either treatment group, which received a daily supplement drink with 200 g of carnosine together with computer cognitive behavior treatment (CCBT), or placebo group. After a six-week follow-up period, the study demonstrated that the carnosine and CCBT group showed significant improvements in fatigue [58].

## 4. Discussion

Carnosine is a natural occurring dipeptide and an over-the-counter food supplement that has been shown to exert multimodal and neuroprotective activity, including the detoxification of free radicals [59], the down-regulation of pro-inflammatory markers [60], as well as the modulation of immune cells (e.g., macrophages and microglia [25,26,61,62]), including the synthesis and the release of neurotrophins such as transforming growth factor beta-1 (TGF-β1) [62].

**Figure 6:**
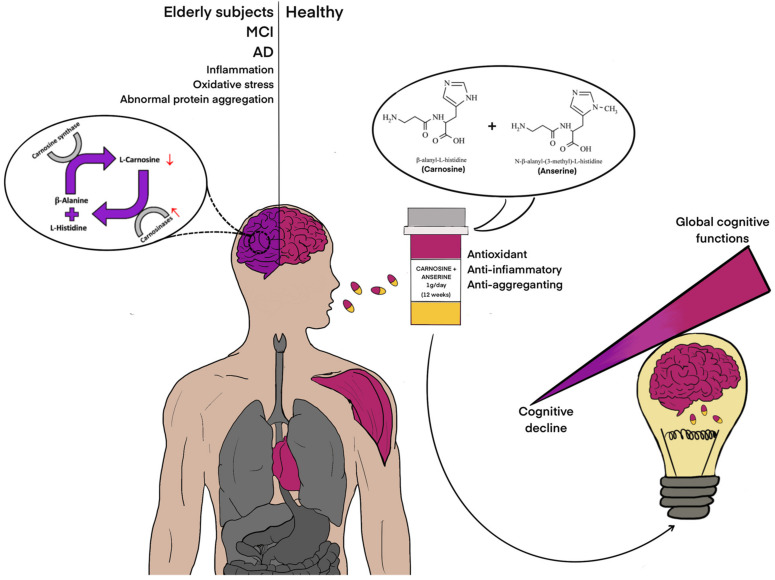
Improvement of global cognitive functions following carnosine/anserine supplementation. Carnosine can be found at mM concentrations at brain level as well as in cardiac and skeletal muscles (indicated in fuchsia). A selective deficit of carnosine and an age-related increase in the serum-circulating carnosinase have been linked to cognitive decline in elderly, MCI and AD subjects (indicated in purple). Red ↑ indicates an increase of carnosinase activity; red ↓ indicates a decrease of carnosine bioavailability. Abbreviations: AD (Alzheimer’s disease); MCI (mild cognitive impairment).

Interestingly, carnosine is able to counteract different factors, such as neuroinflammation, oxidative stress, and the deficit of neurotrophic factors which are strictly connected with aging-related cognitive decline and the risk to develop dementia [37] (Figure 6).

There is evidence that dietary factors may modulate oxidative stress, which in turn play a role on cognitive decline with aging in healthy adults [63]. A review written by Gorelick, including observational epidemiological studies and clinical trials, strongly suggests that inflammation also significantly contribute to cognitive impairment and dementia [64]. Last on the background, it has been demonstrated that immune system dysfunction and the impairment of neurotrophins signaling, such as brain-derived neurotrophic factor (BDNF) and TGF-β1, could promote cognitive decline [65] and neurogenesis [66], while the activation of immune cells (e.g., group 2 innate lymphoid cells) alleviates aging-associated cognitive decline [67].

Starting from the strong preclinical evidence, the therapeutic potential of carnosine to enhance cognition in elderly people as well as in patients suffering of brain-related disorders has been recently considered [52], but the question on the clinical impact of carnosine on cognitive decline still remains open.

We conducted the present systematic review with meta-analysis, in accordance with the PRISMA guidelines coupled to the PICOS approach, to examine the clinical efficacy of carnosine on cognitive function and depressive symptoms in elderly subjects. We first examined the effects of carnosine on cognitive function. When considering all the available studies evaluating the impact of carnosine supplementation on cognitive function, we found that only three trials provided sufficient data to be eligible for quantitative comparison of cognitive decline (Figure 1).

The studies included in our meta-analysis involved partially elderly patients with age-related cognitive decline and MCI patients, a population at high risk of developing AD [46,50,57]. All studies reported, to a various extent, improvements in certain measurements of cognitive status. Another two studies [49,51] conducted on a subgroup sample of Masuoka et al., not included in our meta-analysis, showed similar results as for the main study. It is noteworthy to underline that when restricting the analysis to psychometric tools for the evaluation of global cognitive function (i.e., MMSE and ADAS, specifically), individual studies failed to report significant changes, while an overall effect could have been observed when pooling the results. This observation might have different explanations. First, it may be possible that carnosine might affect specific cognitive functions, perhaps observable in the clinical context with more specific tools rather than a general assessment of cognitive status. However, we were not able to detect clinically-relevant effects of carnosine on verbal memory as assessed by WMS-LM in 2 different clinical trials (Katakura et al. [57], Masuoka et al. [46]). Verbal memory deficits are associated with age-related cognitive decline and, most importantly, with MCI [68]. If we consider the increased effects detected by Masuoka et al. in the APOE4 (+) MCI subjects [69], larger long term (i.e., six months) double-blind, randomized, placebo-controlled trials in amnestic MCI patients are needed to confirm this preliminary evidence of clinical efficacy of carnosine. Second, as individual studies reported improvements in cognitive function of individuals in the intervention groups compared to placebo, but failed showing significant results, it may be possible that larger sample sizes are needed to achieve statistical significance in individual intervention trials.

Most of the studies selected in the present meta-analysis elderly patients received a formula containing 500 mg of carnosine/anserine or 1 g carnosine/anserine. Anserine is a natural derivative of carnosine, usually adopted because it is not cleaved by human carnosinase, which is abundant in human serum and is known to reduce carnosine bioavailability [70]. Anserine and carnosine have equivalent reported physiological functions [7], but the high preclinical evidence on the procognitive effects of carnosine suggest that further clinical studies with carnosine alone (1 g/die) vs. placebo are needed to better understand the therapeutic potential of carnosine against cognitive decline.

Carnosine could prevent and/or counteract the cognitive decline observed in MCI and AD patients through its anti-aggregation activity [71,72]. Insoluble protein aggregates have been found in MCI and AD brain [73], and carnosine might exert its precognitive effects by preventing the transition from Aβ monomers to Aβ oligomers [37]. Furthermore, it cannot be excluded that carnosine can exert its therapeutic potential against cognitive decline, by rescuing BDNF and TGF-β1 signaling [62,74], two neurotrophins whose impairment has been linked to age-related cognitive decline and MCI [65,75,76]. In addition to the well-known antioxidant, anti-inflammatory, and anti-aggregation activities, it has also been shown that carnosine is able to reduce advanced glycation end products (AGEs) and tumor necrosis factor-α (TNF-α) levels in patients with type 2 diabetes mellitus (T2DM) [77]. T2DM is known to be a risk factor for MCI and AD [78], and different neurobiological links have been identified between T2DM and AD such as insulin resistance, low-grade inflammation, increased oxidative stress, and accumulation of AGEs [79,80]. When considering the preliminary evidence of clinical efficacy of carnosine in T2DM patients on insulin resistance, AGEs, and TNF-α, future clinical studies should be conducted in T2DM patients with MCI to better understand the therapeutic potential of carnosine against cognitive decline.

In the present systematic review and meta-analysis, we also examined the effects of carnosine on depressive symptoms, starting from a large body of evidence which shows a strong link between depression and cognitive deficits both in elderly depressed patients and MCI patients [81]. In the present study we failed to find any effect of carnosine supplementation on depressive symptoms, as assessed by different and validated psychometric tools in the elderly, such as the Geriatric Depression Scale (GDS). These results can be explained by considering the high heterogeneity of psychometric tools adopted in the selected studies, as well as the exclusion from these trials of patients with major depressive disorder (MDD). Cognitive dysfunction represents a distinct biological and clinical dimension in MDD that strongly affects psychosocial functioning in MDD patients [82]. A recent study conducted by Araminia et al. [83] found the first evidence of clinical efficacy of L-Carnosine (400 mg twice daily) against affective symptoms as add-on therapy in MDD patients, but the authors did not analyze the impact of the peptide on cognitive symptoms. Further double-blind, randomized, placebo-controlled trials are needed in elderly MDD patients or depressed MCI patients to evaluate the clinical efficacy of carnosine both on cognitive and affective symptoms in MDD.

## 5. Conclusions

A selective deficit of carnosine has been linked to cognitive decline in AD, also promoted by the age-related increase in CN1 activity in the brain. Along this line, different preclinical studies have demonstrated the neuroprotective and procognitive effects of carnosine in experimental models of AD. It is; therefore, expected that carnosine supplementation can improve cognitive function in elderly subjects with age-related cognitive decline as well as in MCI patients with a high risk to develop AD. We conducted the present systematic review with meta-analysis to investigate the therapeutic potential of carnosine against cognitive decline and depressive symptoms in elderly subjects. We found that carnosine/anserine administered for 12 weeks, at a dose of 500 mg^−1^ g/day, improved global cognitive function and verbal memory in the four selected double-blind, randomized, placebo-controlled trials, whereas no effects were detected on depressive symptoms. These data suggest preliminary evidence of the clinical efficacy of carnosine against cognitive decline, both in elderly subjects and MCI patients, although larger and long-term clinical studies are needed in MCI patients (with or without depression) to confirm the therapeutic potential of carnosine.

## Figures and Tables

**Table 1:** PICOS criteria.

PICOS	Description
P (Population)	Men and/or women, adults.
I (Intervention)	Carnosine supplementation (carnosine alone or combined with other treatment).
C (Comparison)	Carnosine supplementation group (carnosine alone or combined with other treatment) versus placebo/control group.
O (Outcomes)	Changes in cognitive function, depressive symptoms, and overall mental health. Long term changes rather than acute effect.
S (Study design)	Systematic review with meta-analysis.

**Table 2:** Main characteristics of the studies included in the systematic review and meta-analysis.

Author and Publication Year	Study Design, Country	Sample Size, Trial Duration	Sex, Age (mean ± SD)	Population Characteristics	Intervention (and Doses)	Measured Outcomes of Interest	NIH Quality Assessment
Szcześniak et al. 2014	Double-blind randomized placebo-controlled trial, Poland	51, 13 weeks	MF, 81.0 ± 7.0 y intervention group, 80.5 ± 7.5 y control group	Nursing home residents, MMSE score >15	1 g of anserine/carnosine (2:1 ratio); once a day	Cognitive function (MMSE, STMS), depressive symptoms (GDS), dementia (CDR)	Good
Cornelli 2010	Double-blind randomized controlled trial, USA	48, 6 months	MF, 75.0 ± 4.2 y intervention group, 74.0 ± 4.9 y control group	Patients with diagnosis of probable AD, MMSE score >21	Formula F (100 mg carnosine and antioxidant compounds: vitamins B, vitamin C and E, coenzyme Q10, beta-carotene, selenium, l-cysteine, Ginko biloba); once per day	Cognitive function (MMSE)	Good
Katakura et al. 2017	Double-blind randomized placebo-controlled trial, Japan	60, 3 months	MF, 60.4 ± 2.1 y intervention group, 65.3 ± 1.6 y control group	Healthy elderly volunteers	1 g of anserine/carnosine (3:1 ratio); twice a day	Cognitive function (MMSE, MCS), Alzheimer’s disease (ADAS), memory (WMS-LM1, WMS-LM2), depressive symptoms (BDI)	Good
Masuoka et al. 2019	Double-blind randomized placebo-controlled trial, Japan	50, 12 weeks	MF, 72.9 ± 8.8 y intervention group, 73.6 ± 6.1 y control group	Outpatients with MCI, MMSE >23	750 mg anserine and 250 mg carnosine; once a day	Cognitive function (MMSE), Alzheimer’s disease (ADAS), dementia (CDR), memory (WMS), depressive symptoms (GDS),	Good
Shirotsuki et al. 2017	Randomized placebo- controlled trial, Japan	72, 6 weeks	MF, 37.88 ± 9.15 y intervention group, 38.35 ± 8.83 y control group	Healthy full-time office workers	Supplement drink with 200 mg carnosine and computerized cognitive behavior therapy; once a day	Mood (POMS)	Fair

Abbreviations: AD (Alzheimer’s disease); ADAS (Alzheimer’s Disease Assessment Scale); AVLT (Auditory Verbal Learning Test); BDI (Beck Depression Inventory); CCBT (computerized cognitive behavior therapy); CDR (Clinical Dementia Rating); CES-D (Centers for Epidemiologic Studies Depression Scale); F (female); GDS (Geriatric Depression Scale); M (male); MCI (mild cognitive impairment); MCS (Mental health Component Summary); MMSE (Mini Mental State Examination); POMS (Profile of Mood Scale); RCT (randomized controlled trial); STMS (Short Test of Mental Status); WMS-LM (Wechsler Memory Scale Logical Memory); y (years).

## Supplementary Materials

The following are available online at https://www.mdpi.com/2227-9059/9/3/253/s1, Supplementary Figure S1: Risk of bias assessment of the included studies according to the Cochrane risk-of-bias tool for randomized trials (RoB-2), Supplementary Figure S2: Funnel plots of studies for cognitive (A), verbal memory (B,C), and depressive symptoms (D) outputs, Supplementary Table S1: Preferred Reporting Items for Systematic Reviews and Meta-Analysis (PRISMA) checklist, Supplementary Table S2: NIH Quality Assessment of Controlled Intervention Studies, Supplementary Table S3: Meta-regression analysis for investigated outcomes.
